# Eradication and Sensitization of Methicillin Resistant *Staphylococcus aureus* to Methicillin with Bioactive Extracts of Berry Pomace

**DOI:** 10.3389/fmicb.2017.00253

**Published:** 2017-02-21

**Authors:** Serajus Salaheen, Mengfei Peng, Jungsoo Joo, Hironori Teramoto, Debabrata Biswas

**Affiliations:** ^1^Department of Animal and Avian Sciences, University of MarylandCollege Park, MD, USA; ^2^Biological Sciences Program – Molecular and Cellular Biology, University of MarylandCollege Park, MD, USA; ^3^Center for Food Safety and Security Systems, University of MarylandCollege Park, MD, USA

**Keywords:** *Staphylococcus aureus*, phenolic compounds, antibiotic resistance, antimicrobials, gene expression

## Abstract

The therapeutic roles of phenolic blueberry (*Vaccinium corymbosum*) and blackberry (*Rubus fruticosus*) pomace (commercial byproduct) extracts (BPE) and their mechanism of actions were evaluated against methicillin resistant *Staphylococcus aureus* (MRSA). Five major phenolic acids of BPE, e.g., protocatechuic, p. coumaric, vanillic, caffeic, and gallic acids, as well as crude BPE completely inhibited the growth of vegetative MRSA *in vitro* while BPE+methicillin significantly reduced MRSA biofilm formation on plastic surface. In addition, BPE restored the effectiveness of methicillin against MRSA by down-regulating the expression of methicillin resistance (*mecA*) and efflux pump (*norA*, *norB*, *norC*, *mdeA*, *sdrM*, and *sepA*) genes. Antibiogram with broth microdilution method showed that MIC of methicillin reduced from 512 μg/mL to 4 μg/mL when combined with only 200 μg Gallic Acid Equivalent (GAE)/mL of BPE. Significant reduction in MRSA adherence to and invasion into human skin keratinocyte Hek001 cells were also noticed in the presence of BPE. BPE induced anti-apoptosis and anti-autophagy pathways through overexpression of *Bcl-2* gene and down-regulation of *TRADD* and *Bax* genes (inducers of apoptosis pathway) in Hek001 cells. In summary, novel and sustainable prophylactic therapy can be developed with BPE in combination with currently available antibiotics, especially methicillin, against skin and soft tissue infections with MRSA.

## Introduction

*Staphylococcus aureus* has historically been a tenacious human pathogen; in recent decades it has become an even more serious threat due to its acquisition of multiple antibiotic resistances (AR) (Wilkinson et al., 2005; Novick, 2008). AR is growing worldwide, and the discovery of new antibiotics is not keeping pace with evolving bacterial resistance; alternative therapeutics is urgently required. Moreover, certain pathogenic *S. aureus* strains known as “superbugs” are exceptionally rigid and quickly develop protective mechanisms against synthetic antibiotics. One common superbug increasingly seen in hospitals and community is methicillin-resistant *Staphylococcus aureus* (MRSA). MRSA is resistant to most antibiotics currently available (Fair and Tor, 2014).

Tang et al. (2011) has reported that MRSA accounts for more than half of all skin and soft tissue infections (SSTIs). In addition, community-acquired-MRSA (CA-MRSA) has also been found in livestock animals, veterinary hospital, and workers (Wulf et al., 2007, 2008). CA-MRSA are recognized as one of common superbugs by multi-locus sequence typing (Ekkelenkamp et al., 2006; Wulf et al., 2008). Though infection with MRSA in hospital setting is being reduced in recent years, the Centers for Disease Control and Prevention (CDC) estimated that the number of MRSA infections in the US exceeds 94,000 per year, and causes more deaths in the US than human immunodeficiency virus and Acquired Immune Deficiency Syndrome (Bancroft, 2007). In addition to hospital settings, CA-MRSA has become a global public health concern (Gardam, 2000; Le Loir et al., 2003; Do Carmo et al., 2004; Gustafson and Wilkinson, 2005; Kennedy et al., 2008; Pu et al., 2009). Because of these emergent issues, development of alternative strategies to combat MRSA infection is of pressing need.

The seeds and skins of berries contain polyphenols, phytosterols, tocopherols, fiber, protein, and biotin (Puupponen-Pimiä et al., 2005, 2008; Salaheen et al., 2014, 2015). Major components of polyphenols include anthocyanins, procyanidins, and hydroxycinnamates. Procyanidins are a class of polymeric polyphenolics and can contain catechin or epicatechin (Djilas et al., 2009). Our recent HPLC/high mass accuracy TOF mass spectrometry analysis indicated that major phenolic compounds in berry pomace (byproduct) include but are not limited to flavan, glucuronides, quinolones, catechol, coumarin, phenols, tannins, quercetin, vanillic acid, caffeic acid, ellagic acid, and gallic acid (Salaheen et al., 2016). Flavans are a type of anthocyanidin pigments with antimicrobial activity against several pathogenic bacteria (Sasaki et al., 2007; Yao et al., 2011). Antimicrobial properties in glucuronides, catechol, and phenols have also been reported (Salem and Saleh, 2002; Puupponen-Pimiä et al., 2008). Quinolones have antimicrobial activity and anti-quorum sensing activity against *Pseudomonas* (Rasmussen and Givskov, 2006). Alves et al. (2013) has reported that acid derivatives including vanillic acid, caffeic acid, ellagic acid, and gallic acid from mushrooms were able to synergistically inhibit MRSA.

In this study, we aim to evaluate the function of phenolic blueberry and blackberry pomace extracts (BPE) in restoring the antibacterial activity of methicillin by complete inhibition of MRSA cells both vegetative and biofilm formation through alteration of physical, biochemical, and transcriptome profile.

## Materials and Methods

### Preparation of Pomace Extracts

Bioactive phenolic extracts from blackberry (*Rubus fruticosus*) and blueberry (*Vaccinium corymbosum*) pomaces were prepared according to the protocol previously described (Salaheen et al., 2014). Total phenolic contents in each extract was determined using spectrophotometric method (Singleton et al., 1999). Total phenolic content was expressed as Gallic Acid Equivalent (GAE). Berry Pomace Extract (BPE) was prepared by combining blackberry and blueberry pomace extract at 1:1 ratio. The pH ranges of the crude extracts were 4.5–5 and pH of the extracts varied with the methods of treatment and extraction.

### Bacterial Strains, Growth Condition, Antimicrobial Susceptibility, and Methicillin Rescue Assay

Methicillin resistant *Staphylococcus aureus* COL (SCCmec type I; gift from Dr. Steven Ricke, University of Arkansas; complete genome available from http://www.genome.jp/dbget-bin/www_bget?gn:T00225) was used in the current study for *in vitro* assays. Strain COL is resistant to oxacillin, methicillin, and tetracycline, possesses a non-functional *mecI*-*mecR1* system, and β-lactamase deficient (De Lencastre and Tomasz, 1994; De Lencastre et al., 1999). Bacterial cells were grown in Cation Adjusted Muller Hinton Broth (CAMHB; Clinical and Laboratory Standards Institute, [CLSI], 2013) at 37°C for 24 h with shaking at 120 rpm.

Minimum Inhibitory Concentrations (MIC) and Minimum Bactericidal Concentration (MBC) of BPE, and other purified phenolic compounds including Protocatechuic acid, Taxifolin, Myricetin, Quercetin, P. coumaric acid, Gallic acid, Vanillic acid, and Caffeic acid were determined using broth micro-dilution method described previously with some modifications (Salaheen et al., 2015). In brief, BPE concentration starting from 4092 μg GAE/mL to 0.0 μg GAE/mL with a twofold dilution were added to CAMHB to a final volume of 990 μL. Ten microliter bacterial suspension containing 5 × 10^5^ CFU/mL was added to 24-wells plates followed by incubation at 37°C with shaking at 120 rpm for 24 h. After incubation, bacterial suspensions were serially diluted in PBS and plated on Baird–Parker agar medium (BP, Himedia, India). Growth inhibition and all other assays were repeated at least three times with three biological replicates.

Antibiotic (methicillin) rescue assay was also carried out in CAMHB with 2% NaCl. BPE and/or methicillin containing broth was added to 96-well culture plates (Greiner Bio-One CellStar, NC) with concentration ranging from 0 to 400 μg GAE/mL of BPE and concentration ranging from 0 to 512 μg/mL of methicillin. Plates were incubated for 24 h at 37°C followed by drop-plating on BP.

The dose dependent responses of BPE and/or methicillin were analyzed using the Nonlinear Curve Fitting Function of Microcal Origin 7.5 (Microcal Software Inc., Northampton, MA). The sub-lethal dose (SLT_2LOG_) at which microbial cell numbers were reduced by ∼2 log compared with the control was determined following the method described by Ahn et al. (2014). The SLT_2LOG_ was determined as combination of 25 μg GAE/mL BPE with 4.0 μg/mL of methicillin in CAMHB against MRSA strain.

### Physicochemical and Virulence Properties of MRSA Treated with Sub-lethal Dose of BPE (SLT_2LOG_)

Physicochemical properties of bacterial cells, e.g., cell surface hydrophobicity and auto-aggregation, were evaluated following the methodologies previously described (Rosenberg, 1984; Del Re et al., 2000; Peng et al., 2015c). Virulence properties of MRSA strains, e.g., serum agglutination and hemolysis activity on sheep red blood cells (RBC) (Lampire, Everett, PA, USA) and single human donor RBC (Innovative Research, MI) were determined following the methods described previously by Jiang et al. (2007). MRSA was grown in CAMHB with 2% NaCl in either presence of only 4.0 μg/mL of methicillin or presence of predetermined SLT_2LOG_ of inhibitors (25 μg GAE/mL BPE and 4.0 μg/mL of methicillin) at 37°C for 18 h. The harvested MRSA cells were used to extract RNA with ZR Bacterial RNA MiniPrep kit (Zymo Research, San Diego, CA, USA) for quantitative RT-PCR assay. Serum agglutination test was carried out with BactiStaph Latex Test Kit (Ref: R21144, Remel, Lenexa, KS, USA) and the time required for complete agglutination was measured with stopwatch (VWR, Radnor, PA, USA). Hemolysis activity of MRSA was determined according to the protocol described by Jiang et al. (2007).

### Adhesion and Invasiveness Assay

Adhesion and invasion ability of MRSA strains were determined under two conditions: induction of several concentrations of BPE and methicillin combination treatments during infection (bacterial inoculum and treatments concurrently added on host cell monolayer; representing preventive state) and the same range of treatments after infection (treatments added after 1 h infection of host cell monolayer with bacterial inoculum; representing therapeutic state). A concentration of 4 μg/mL methicillin was used in combination with 0, 50, 100, 200, 400, and 800 μg GAE/mL of BPE as the various treatment groups. The adhesion and invasion of human keratinocyte HEK001 (ATCC CRL 2404^TM^) cells by MRSA strains was carried out following the method previously described (Salaheen et al., 2014; Peng et al., 2015b). To simulate the therapeutic state, treatments containing 4 μg/mL methicillin in conjunction with 0, 50, 100, 200, 400, and 800 μg GAE/mL of BPE were used on infected cell monolayer at 37°C for 1 h.

### Effects of BPE on Cultured HEK001 Cells

Viability of cultured HEK001 cells in the presence of various concentration of BPE was measured following the method described previously with modifications (Strober, 2001). HEK001 cell monolayers were washed and 1 mL of fresh Keratinocyte-SFM medium containing 5 ng/mL epidermal growth factor (EGF) and various concentrations of BPE (0, 50, 100, 200, 400, and 800 μg GAE/mL) were placed in triplicate wells followed by incubation for 2 h at 37°C and viability assay was carried out with 0.4% Trypan Blue solution (Sigma, MO). Treated HEK001 cell monolayers were also imaged with Nexus6 (Google Play Store) and the images were processed in Microsoft Office Picture Manager (Version 2010). Treated HEK001 cell monolayers were also used to extract RNA for quantitative RT-PCR assay.

### Synergistic Effect of BPE and Methicillin on MRSA Growth and Biofilm Formation

To prepared biofilms, MRSA was inoculated at approximately 5 × 10^5^ CFU/mL in 6-well plates (Corning, New York, NY, USA) containing 22 mm × 22 mm plastic slides (VWR) and CAMHB with 2% NaCl at 37°C for 96 h without shaking. CAMHB containing 2% NaCl with 4 μg/mL methicillin was combined with MIC or double MIC of BPE and incubated at 37°C under shaking at 120 rpm for 1 and 24 h. For numerical analysis, MRSA cells were recovered with sterile cell scraper (VWR, PA) from the plastic surface and plated on BP.

For visualization, treated biofilms on plastic slides were stained with FilmTracerTM LIVE/DEAD Biofilm Viability Kit (Life Technologies, OR). Slides were viewed at room temperature using a Zeiss AxioObserver. Z1 inverted microscope, and images were acquired using the Zeiss Axiovision Rel 4.6 software with the Zeiss Axiocam HRC camera. All exported images were processed in Microsoft Office Picture Manager (Version 2010). RNA was also extracted from the bacterial cells of treated MRSA biofilms on plastic slides and used for quantitative RT-PCR assay.

### Measurement of Transcriptomic Changes of MRSA and HEK001 Cells Treated with BPE and Methicillin

To measure the expression of MRSA and HEK001cell genes in the presence of BPE, quantitative RT-PCR assay was carried out in three different sections of this study: (i) expression of virulence and antimicrobial resistance mediatory genes of MRSA, (ii) expression of genes involved in biofilm formation of MRSA, and (iii) expression of the genes related to cellular growth and apoptosis of host HEK001 cells. RNA extraction procedures have been described above. QScript cDNA Supermix (QuantaBio, Beverly, MA, USA) was used from cDNA synthesis and qRT-PCR was performed in Eco^TM^ (Illumina, San Deigo, CA, USA) according to the protocol previously described (Peng et al., 2015c).

### Statistical Analysis

All data were analyzed using the Statistical Analysis System software (SAS, Institute Inc., Cary, NC, USA). One-way analysis of variance (ANOVA) was used, followed by Tukey’s test to determine significant differences among treatments at *p* < 0.05.

## Results

### Inhibition of MRSA Growth with Phenolics

The inhibitory effects of various phenolics and their respective MBC have been listed in **Table 1**. We found that the MBC of the tested phenolic acids including Protocatechuic acid, P. coumaric acid, Gallic acid, Vanillic acid, and Caffeic acid inhibited the growth of MRSA in CAMHB liquid culture at concentrations ranging from 0.512 to 2.046 mg GAE/mL with P. coumaric acid being the most effective>1.028 mg GAE/mL of combination of these five phenolic acids were required for inhibiting the growth of MRSA. Flavonoids, e.g., Taxifolin, Myricetin, and Quercetin, failed to demonstrate any growth inhibitory effect up to 4.092 mg GAE/mL. Crude BPE exhibited the highest inhibitory effect against MRSA, the MBC and MIC were observed 0.512 mg GAE/mL and 0.400 mg GAE/mL, respectively.

**Table 1:** Inhibition of *Staphylococcus aureus* COL growth in presence of multiple phenolics.

Treatments	Concentration (μg GAE/mL)^#^					
	**4092**	**2046**	**1028**	**512**	**256**	**128**

Protocatechuic acid		+	++	++	++	++
Taxifolin	++	++	++	++	++	++
Myricetin	++	++	++	++	++	++
Quercetin	++	++	++	++	++	++
P. coumaric acid				++	++	++
Gallic acid			++	++	++	++
Vanillic acid			++	++	++	++
Caffeic acid			++	++	++	++
Combination^∗^			++	++	++	++
BPE				+	++	++

*‘++’ represents >300 cfu/mL; ‘+’ represents <300 cfu/mL ^#^Concentrations were converted to GAE unit ^∗^Combination included Protocatechuic acid. P. coumaric acid, Gallic acid, Vanillic acid, and Caffeic acid*

### Synergistic Effect of BPE with Methicillin against MRSA

**FIGURE 1:**
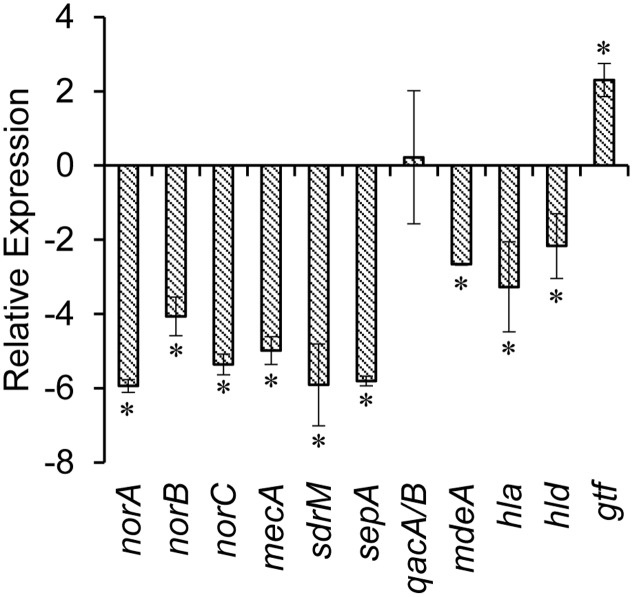
**Relative expression of virulence genes of methicillin resistant *Staphylococcus aureus* (MRSA) treated with SLT_2LOG_ of BPE and methicillin**. ^∗^indicates significantly increased or decreased relative expression of genes at *p* < 0.05.

The synergistic inhibitory effects of BPE and methicillin against the MRSA have been illustrated in **Table 2**. MBC of methicillin against MRSA was >512 μg/mL, whereas conjunction with various concentrations of BPE minimized the MBC of methicillin to as low as 4 μg/mL which is below the concentration range suggested by the CLSI for MRSA. We determined the SLT_2LOG_ (treatment at which MRSA numbers were reduced by 2 logs compared with control group) to be as the combination of 25 μg GAE/mL BPE with 4 μg/mL of methicillin and used this treatment for further studies. We observed that the combination of 200 μg GAE/mL BPE with 4 μg/mL of methicillin in CAMHB broth reduced MRSA by >4 logs compared to the treatments of 200 μg GAE/mL BPE or 4 μg/mL of methicillin in CAMHB individually. To determine the reactivation of effect of methicillin through efflux pump genes alteration, we checked the differential expression levels in the multi-drug resistance (MDR) efflux pump genes in MRSA in the presence of SLT_2LOG_ (**Figure 1**). For example, the expression of MDR efflux pump genes, e.g., *norA*, *norB*, *norC*, *mdeA*, *sdrM*, and *sepA* were significantly down-regulated. More specifically, SLT_2LOG_ also reduced the expression of methicillin resistance gene, *mecA*. However, SLT_2LOG_ did not alter the expression of *qacA/B* gene, and stimulated *gtf* gene.

**Table 2:** Sensitization of *S. aureus* COL to Methicillin in presence of BPE.

	Methicillin (μg/mL)										
		512	256	128	64	32	16	8	4	0
**BPE (μg GAE/mL)**	400									++


	200								+	++


	100				+	++	++	++	++	++


	50	+	++	++	++	++	++	++	++	++
	25	++	++	++	++	++	++	++	++	++
	12.5	++	++	++	++	++	++	++	++	++
	6.25	++	++	++	++	++	++	++	++	++
	0	++	++	++	++	++	++	++	++	++

*‘++’ represents >300 cfu/mL; ‘+’ represents <300 CFU/mL*

### Alteration of Physicochemical Properties and Expression of Virulence Genes of MRSA in the Presence of Sub-lethal Treatment (SLT_2LOG_) of BPE and Methicillin Combination

The effects of the SLT_2LOG_ on the physicochemical properties and virulence factors of MRSA have been shown in **Table 3**. In the presence of SLT_2LOG_ BPE and methicillin, auto-aggregation capability of MRSA increased significantly. Untreated bacterial cells showed lower auto-aggregation, 8.84 ± 1.64% whereas in presence of SLT_2LOG_ the value was increased to 15.18 ± 1.27% (*p* < 0.05). A co-occurrence was noticed between the phenomenon of increased auto-aggregation in MRSA to enhanced expression of *gtf* gene which codes for polysaccharide synthase playing important roles in exopolysaccharide formation (Deutsch et al., 2008). SLT_2LOG_ of BPE and methicillin combination also reduced the hemolytic property of MRSA; hemolysis of sheep and human RBCs by MRSA were reduced to 59.50 ± 8.36%, and 73.50 ± 26.07%, respectively, in the presence of SLT_2LOG_. The expression of α-hemolysin (*hla*) and δ-hemolysin (*hld*) genes were also significantly down-regulated with SLT_2LOG_ (**Figure 1**)_._ However, cell surface hydrophobicity and serum agglutination capability of MRSA were not altered significantly with SLT_2LOG_.

**Table 3:** Physicochemical properties and mechanical behaviors of *S. aureus* COL treated with sub-lethal concentration (SLC_2LOG_) of berry pomace extract (BPE) and methicillin.

Treatments	Auto-aggregation (%)	Hydrophobicity (%)	Serum Agglutination (s)	Hemolysis (%) Sheep RBC	Hemolysis (%) Human RBC
Control	8.84 ± 1.64^?,a^	69.99 ± 0.65^a^	48.09 ± 11.08^a^	100^a^	100^a^
BPE	15.18 ± 1.27 ^b^	66.71 ± 5.72^a^	52.30 ± 6.83^a^	59.50 ± 8.36^b^	73.50 ± 26.07^b^

^?^*Means with different letters within an individual column (a-c) are significantly different at *p* < 0.05*.

### Role of SLT_2LOG_ Dose of BPE and Methicillin Combination on Human Keratinocyte Cells-MRSA Interactions

**FIGURE 2:**
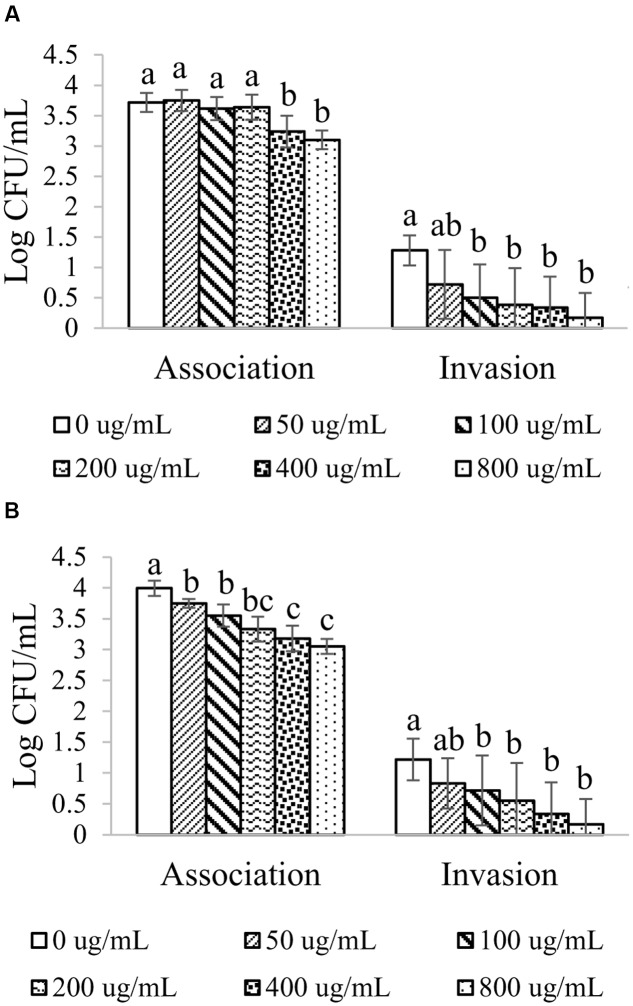
**Adhesion and invasion ability of MRSA to HEK001 in the presence of various concentrations of BPE and BPE combined with 4 μg/mL of methicillin during infection** **(A)** and post infection **(B)**. Means with different letters **(A–D)** are significantly different (*p* < 0.05).

Adherence and invasion of human keratinocyte HEK001 cells with MRSA were performed under several concentrations of BPE in combination with methicillin during treatment and post-infection phase (**Figure 2**). In the presence of BPE, adherence of MRSA to HEK001 cells were decreased at a dose-dependent manner. Significant reduction in the adherence of MRSA to HEK001 was observed at the concentration from 400 μg GAE/mL of BPE and beyond (*p* < 0.05). Numerical reduction in MRSA adherence to HEK001 cells was noticed with the lowest concentration, e.g., 100 μg GAE/mL of BPE (**Figure 2**). Invasion of MRSA into HEK001 cells was also plummeted at a dose-dependent manner (**Figure 2A**). Significant difference in the invasion of MRSA into HEK001 was observed at the concentration of BPE starting from 100 μg GAE/mL (**Figure 2A**).

A dose-dependent decrease in the adherence of MRSA to HEK001 cells was also observed when infected HEK001 cells were treated with BPE (**Figure 2B**). Significant difference in the adherence of MRSA to HEK001 (0.3–1.5 logs) was observed at the concentration of BPE from 50 μg GAE/mL and beyond (*p* < 0.05). Invasion of MRSA into HEK001 cells was reduced by 0.3–1 log depending on the concentration of BPE; significant difference in the invasion of MRSA into HEK001 cells was observed at the concentration of BPE starting from 100 μg GAE/mL.

### Effect of BPE on Human Keratinocyte (HEK001) Cell Viability

**FIGURE 3:**
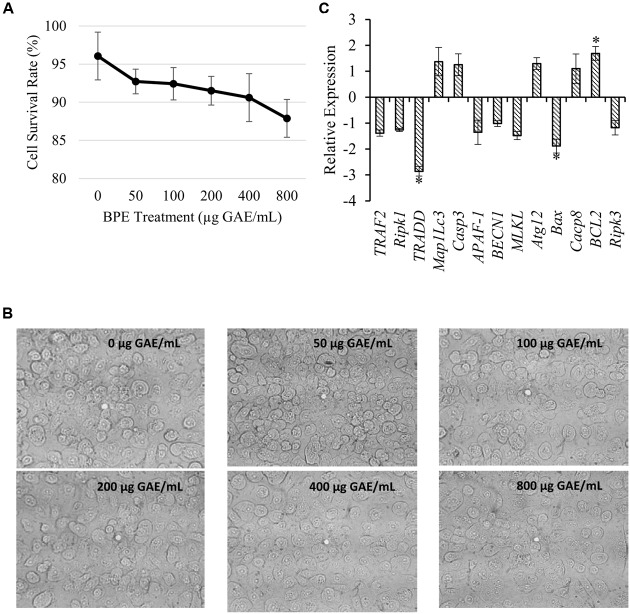
**Effect of various concentrations of BPE combined with 4 μg/mL of methicillin on MRSA-cultured HEK001 cell interactions**. **(A)** Cell survival rate at various concentrations of BPE; **(B)** Visualization of cell morphology in the presence of BPE; **(C)** Expression of the genes related to HEK001 cell growth and apoptosis after treatment with 200 μg GAE/mL of BPE combined with 4 μg/mL of methicillin. ^∗^indicates significant differential value compared to the BPE (–) Methicillin 4 μg/mL (+) group at *p* < 0.05.

Trypan blue exclusion assay indicated a dose-dependent reduction in the viability of adherent HEK001 cell (**Figure 3A**). The percentages of 96.06, 92.72, 92.42, 91.51, 90.61, and 87.88 of adherent HEK001 cells to the plastic surfaces were remained viable after 2 hr treatment with 0, 50, 100, 200, 400, and 800 μg GAE/mL of BPE, respectively (**Figure 3A**). Usually, human cultured keratinocytes are large, epithelial, round, adherent cells growing as a confluent monolayer and after 2 h exposure to BPE, neither substantial changes in cell morphology, nor any detachment from the surface were noticed microscopically (**Figure 3B**). However, a slight dehydration was observed with increased concentrations of BPE. Investigation into the expression of HEK001 genes active in necrosis, apoptosis, and autophagy pathways indicated that treatment with BPE only induced the expression of *Bcl-2* (inhibitor to apoptosis and autophagy pathways) by ∼ 2 while down-regulating *TRADD* and *Bax* genes (inducers of apoptosis pathway) by two- to threefold (**Figure 3C**).

### Anti-biofilm Formation Effect of BPE in Combination with Methicillin

**FIGURE 4:**
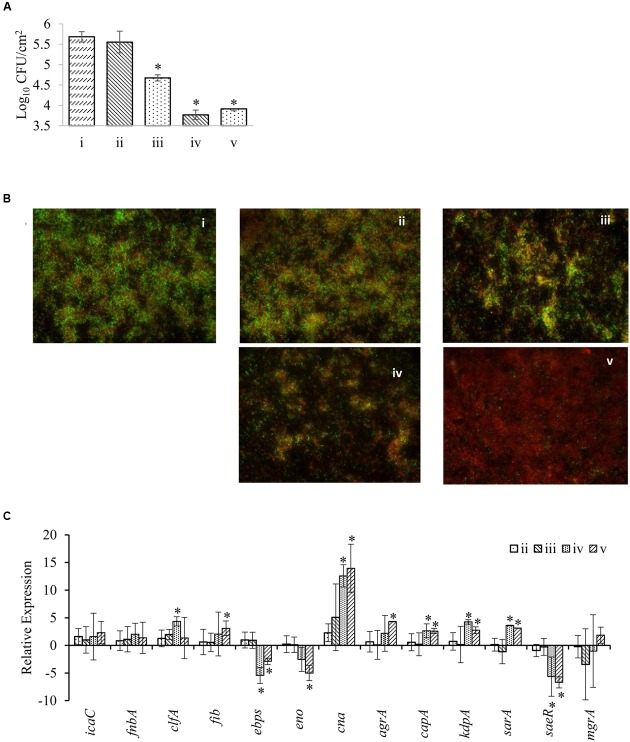
**Effect of BPE combined with 4 μg/mL of methicillin on MRSA biofilm formation**. **(A)** Inhibition of MRSA cells forming biofilm on plastic surface; **(B)** fluorescence microscopy of MRSA biofilms in the presence of BPE; **(C)** Expression of the genes related to biofilm formation in MRSA. Treatments: (i) BPE (–) Methicillin 4 μg/mL (+); ii, BPE (MIC) Methicillin 4 μg/mL (+) 1 h; (iii) BPE (2x MIC) Methicillin 4 μg/mL (+) 1 h; (iv) BPE (MIC) Methicillin 4 μg/mL (+) 24 h; (v) BPE (2x MIC) Methicillin 4 μg/mL (+) 24 h. ^∗^indicates significant differential value compared to the BPE (–) group at *p* < 0.05.

Effect of BPE (MIC and 2x MIC) in combination with 4 μg/mL methicillin on preformed MRSA biofilms has been documented (**Figure 4**). Treatment with only 2x MIC of BPE (but not MIC of BPE) combined with 4 μg/mL methicillin for 1 h treatment significantly reduced the number of MRSA cells (∼1 log) in preformed biofilm on plastic surface (**Figure 4A**). We also observed in the same assay that the longer period of treatments with both MIC and 2x MIC of BPE (24 hr) caused significant reductions (1.5–2.0 logs) in the viability of biofilmed MRSA. Microscopic observation using SYTO 9 green fluorescence dye (stains live biofilm cells) and propidium iodide (stains cells with injured outer membrane) also confirmed these findings (**Figure 4B**). Highest inhibition of MRSA biofilm was observed on treatment with 2x MIC of BPE combined with 4 μg/mL methicillin after 24 h. MIC of BPE with 4 μg/mL methicillin resulted in MRSA membrane injury (visible red and orange MRSA cells) but no significant alteration in the MRSA viability on agar medium. The expressions of 13 genes of MRSA that are involved in biofilm formation were compared and the annotations of these genes are described in **Supplementary Table S1**. Differential expression was observed with treatment among 10 of these genes: significant up-regulation in seven (*clfA*, *fib*, *cna*, *agrA*, *capA*, *kdpA*, and *sarA*) and down-regulation in three (*ebps*, *eno*, and *saeR*) compared to non-treatment group. Expression of *cna*, *clfA*, *fib*, *agrA*, *capA*, *kdpA*, and s*arA* was 3–15 fold up-regulated at 24 h of treatment, with *cna* being the most up-regulated gene (**Figure 4C**). On the other hand, *ebps*, *eno*, and *saeR* exhibited 3–5 fold down-regulations at 24 h of treatment. No significant differential expression of any gene was observed after 1 h treatment with up to 2x MIC concentration of BPE.

## Discussion

Due to the lack of success in developing new antibiotics by pharmaceutical companies and the common trend of bacterial mechanism in gaining resistance against currently available antibiotics through natural selection, the World Health Organization has warned that we are heading toward a “post-antibiotic era” (Aryee and Price, 2015). Therefore, recent researches have been directed towards finding new antimicrobials and novel strategies to combat multi-drug resistant bacterial pathogens. In this study, we have tested the function and mechanism of BPE in restoring the antibacterial activity of methicillin against MRSA.

Polyphenols, secondary metabolites in higher plants, are abundantly found in plant tissues mainly as glycosides and/or complex polymerized compounds (Puupponen-Pimiä et al., 2005, 2008; Peng et al., 2015a). The seeds and skins of fruits, particularly berries, contain polyphenols, phytosterols, tocopherols, fiber, protein, and biotin (Salaheen et al., 2014, 2015, 2016). Our recent HPLC/high mass accuracy TOF mass spectrometry analysis indicated that major phenolic compounds in blackberry and blueberry pomace, include but are not limited to flavan, glucuronides, quinolones, catechol, coumarin, phenols, tannins, quercetin, vanillic acid, caffeic acid, ellagic acid, and gallic acid and most of them are antibacterial (Alves et al., 2013; Salaheen et al., 2016). In this study, we confirmed that BPE from both blueberry and blackberry pomace are capable of complete inhibition of MRSA. In addition, findings from this study indicate that BPE compounds perform better synergistically which opens further research options to determine appropriate ratios of these compounds to be used against MRSA. Altered auto-aggregation of MRSA after treatment with BPE and methicillin indicated alteration of cell membrane and consequent rupture of cells followed by cell-death (Salaheen et al., 2014).

Our results indicated that BPE possess the capability to restore the effective antibacterial activity of currently ineffective antibiotics, especially methicillin against MRSA. Though actual mechanism of reactivation of antibiotics have not been documented but our data agreed with previous findings (Stapleton et al., 2004; Aiyegoro and Okoh, 2009; Chung et al., 2011). We found significant down-regulation of MDR efflux pump genes, e.g., *norA*, *norB*, *norC*, *sdrM* and *sepA*, and more specifically methicillin resistance mediatory *mecA* gene in the presence of BPE. Exposure to methicillin inactivates the high-binding-affinity Penicillin Binding Proteins (PBP) that are normally present in *S. aureus*. However, a novel PBP2a protein, encoded by *mecA* gene and normally present in MRSA, displaying a low affinity for methicillin takes over the synthesis of peptidoglycan which permits the MRSA to grow (Chambers, 2001). We hypothesize that the differential expression of *mecA* and MDR efflux pump genes in the presence of BPE is possible mechanism of methicillin reactivation against MRSA. Though sufficient reports are unavailable on the direct implication of efflux pump genes on methicillin resistance in MRSA, these genes are highly expressed in MRSA isolates (Jang, 2016). So, in this study, we included efflux pump genes in addition to the major methicillin resistance gene, *macA*.

In addition to the inhibition of the growth of vegetative MRSA cells, BPE alone and BPE in combination with methicillin significantly reduced the biofilmed MRSA. Biofilm formation behavior of *S. aureus* that are responsible for a variety of diseases in humans (Kskiwiecin et al., 2015). Up-regulation of several genes involved in biofilm formation was observed in the presence of BPE and methicillin. These include major component of quorum sensing in *S. aureus*: accessory gene regulator (*agrA*), capsular polysaccharide regulatory elements (*capA*, *sarA, kdpA*), and microbial surface components recognizing adhesive matrix molecules (*cna, fib, and clfA*). These findings indicate compensatory and stress response in MRSA biofilm in presence of BPE and methicillin. Membrane and slime layer disruption can be considered the major mechanism of BPE to reduce MRSA biofilm on plastic surface (Federman et al., 2016). Further, alteration of other virulence properties, e.g., reduction of RBC hemolysis and reduced infection rate to HEK001 cells were also observed in the presence of BPE.

Skin infections with MRSA often induce excess host inflammation due to cytokines produced by host and exotoxins secreted by the bacterial cells, which in turn aggravate the pathogenesis of the disease (Montgomery et al., 2013; Sharma-Kuinkel et al., 2013). Anti-inflammatory effects of phenolics from BPE would potentially be able to limit the inflammatory process induced by MRSA infection (Lechner et al., 2004). We observed induction of anti-apoptosis and anti-autophagy pathways in the presence of BPE through over-expression of *Bcl-2* gene while down-regulation of *TRADD* and *Bax* genes in HEK001 cells. Interesting to notice that HEK001 cells survival rate in the presence of various concentrations of BPE is not consistent with anti-apoptosis and anti-autophagy genes’ expression. This indicates apoptosis and autophagy independent pathways are responsible for the reduced survival of HEK001 in the presence of BPE. Anti-apoptosis and anti-autophagy pathways in MRSA infected HEK001 cells followed by BPE treatment may play important prophylactic roles in skin and soft tissue infections. At a first glance the MBC value of BPE against MRSA seems high. However, in conditions like skin and soft tissue infections with MRSA, BPE can be used in dressing materials and in that case, the effective concentration will be feasible to use (Muthaiyan et al., 2012).

## Conclusion

Our findings suggest that phenolic extracts of blackberry and blueberry pomaces have high potential to enhance activity of current antibiotic regime, especially methicillin against MRSA. In addition, novel prophylactic substitutes can be developed from the crude extracts of blackberry and blueberry pomaces/fruits for targeting the altered pathogenicity and inhibit the growth of MRSA in both vegetative cells as well as in preformed biofilm and control infection with *S. aureus* regardless its antibiotic resistance patterns.

## Author Contributions

SS contributed to design of the work, performing experiments, interpretation of data for the work, responsible for the integrity of the work as a whole, drafting the manuscript, and revising the work. MP contributed in conducting part of the experiments, responsible for the accuracy of the work, revising the manuscript, and the final approval of the version to be published. JJ contributed to design of the work, performing part of the experiments, interpretation of data for the work, and ensuring the integrity of the work. HT contributed in performing part of the experiments, and the acquisition and analysis of data for the work. DB contributed to the conception and design of the work, ensuring that questions related to the accuracy or integrity of any part of the work are appropriately investigated and resolved, critically revising the final approval of the version to be published, and responsibility for the integrity of the work as a whole.

## Conflict of Interest Statement

The authors declare that the research was conducted in the absence of any commercial or financial relationships that could be construed as a potential conflict of interest.

## Supplementary Material

TABLE S1 **Biofilm formation genes and primers for MRSA**.
