# Supplementary material for: Eradication and Sensitization of Methicillin Resistant Staphylococcus aureus to Methicillin with Bioactive Extracts of Berry Pomace
Source: Front Microbiol. 2017 Feb 21;8:253. doi: 10.3389/fmicb.2017.00253 (PMC5319404; doi:10.3389/fmicb.2017.00253)
Supplement: TABLE S1 — Biofilm formation genes and primers for MRSA. [file Table_1.docx]

| Seq name | Seq 5’-3’ | Reference |
| --- | --- | --- |
| norA-F | GACATTTCACCAAGCCATCAA | Kwak et al., 2013 |
| norA-R | TGCCATAAATCCACCAATCC |  |
| norB-F | GCTACACCATCAACAGATACAGCAA |  |
| norB-R | ACTCAATGCGACGCCAAA |  |
| norC-F | TGGGTTGGAGATGGATTTTC |  |
| norC-R | ACAATTAGCCCTGCAACGTC |  |
| 16S rRNA MRSA-F | CCAGCAGCCGCGGTAAT | Patel et al., 2010 |
| 16S rRNA MRSA-R | CGCGCTTTACGCCCAATA |  |
| mecA-F | AACCGAAGATAAAAAAGAACC |  |
| mecA-R | GTCCGTAACCTGAATCAGC |  |
| mdeA-F | CTTTCAGGTTACCTTGTTGAATATTTAAAC |  |
| mdeA-R | ATCAATAGGTACTTTAATTGTAGTTCCAAC |  |
| mepA-F | ATGGTATAGGTTTCTTGTTTACTGGTATG |  |
| mepA-R | AATGATAATTGCACCTTGTAAAATGGC |  |
| sdrM-F | CAACATGGCATTGGTTATTCTAC |  |
| sdrM-R | ACAGCTGTTGGTTTAATAAAGC |  |
| sepA-F | GAAGTATGTACGATAACCTATTATATTATGGC |  |
| sepA-R | AAAGTCGCGCCTCTAAAATATGC |  |
| qacA/b-F | GGTGCTTTAATAATGCC |  |
| qacA/B-R | CCAGTCCAATCATGCCTGC |  |
| icaC-F | CTTGGGTATTTGCACGCATT | Atshan et al., 2013 |
| icaC-R | GCAATATCATGCCGACACCT |  |
| fnbA-F | AAATTGGGAGCAGCATCAGT |  |
| fnbA-R | GCAGCTGAATTCCCATTTTC |  |
| clfA-F | ACCCAGGTTCAGATTCTGGCAGCG |  |
| clfA-R | TCGCTGAGTCGGAATCGCTTGCT |  |
| fib-F | CGTCAACAGCAGATGCGAGCG |  |
| fib-R | TGCATCAGTTTTCGCTGCTGGTTT |  |
| ebps-F | GGTGCAGCTGGTGCAATGGGTGT |  |
| ebps-R | GCTGCGCCTCCAGCCAAACCT |  |
| eno-F | TGCCGTAGGTGACGAAGGTGGTT |  |
| eno-R | GCACCGTGTTCGCCTTCGAACT |  |
| cna-F | AATAGAGGCGCCACGACCGT |  |
| cna-R | GTGCCTTCCCAAACCTTTTGAGCA |  |
| agrA-F | AAAGTTGCAGCGATGGATTT | Zhao et al., 2010 |
| agrA-R | ATGGGCAATGAGTCTGTGAG |  |
| capA-F | CAGTTAAAGTCGCACCAA |  |
| capA-R | GAACCCAATACAGGCAAT |  |
| kdpA-F | ATTGTTCGGTTTATTGTCC |  |
| kdpA-R | CATCATACTGCCCATTTCT |  |
| sarA-F | GACATACATCAGCGAAAA |  |
| sarA-R | TACGTTGTTGTGCATTAA |  |
| saeR-F | AAGTGGCGACCATTACAT |  |
| saeR-R | CATTATTGCCTCAAATACGT |  |
| mgrA-F | AGTACAATCTAACATACC |  |
| mgrA-R | TTGCGATAAAGAAGAAGC |  |
| hla-F | AATGAATCCTGTCGCTAATGCCGC | Moisan et al., 2006 |
| hla-R | CTGAAGGCCAGGCTAAACCACTTT |  |
| hld-F | TAATTAAGGAAGGAGTGATTTCAATG |  |
| hld-R | TTTTTAGTGAATTTGTTCACTGTGTC |  |
| gtf-F | TGGTGACGCCGAAGGACTC | Deutsch et al., 2010 |
| gtf-R | GCAGCACGAGCAGGAACAC |  |

Atshan, S. S., Shamsudin, M. N., Karunanidhi, A., van Belkum, A., Lung, L. T., Sekawi, Z., Nathan, J. J., Ling, K. H., Seng, J. S., Ali, A. M., Abduljaleel, S. A., and Hamat, R. A. (2013). Quantitative PCR analysis of genes expressed during biofilm development of methicillin resistant *Staphylococcus aureus* (MRSA). *Infect. Genet. Evol.* 18, 106-112.

Deutsch, S. M., Le Bivic, P., Hervé, C., Madec, M. N., LaPointe, G., Jan, G., Le Loir, Y., and Falentin, H. (2010). Correlation of the capsular phenotype in propionibacterium freudenreichii with the level of expression of *gtf*, a unique polysaccharide synthase-encoding gene. *Appl. Environ. Microbiol.* 76(9), 2740-2746.

Kwak, Y. G., Truong-Bolduc, Q. C., Bin Kim, H., Song, K. H., Kim, E. S., and Hooper, D. C. (2013). Association of *norB* overexpression and fluoroquinolone resistance in clinical isolates of *Staphylococcus aureus* from Korea. *J. Antimicrob. Chemother.* 68(12), 2766-2772.

Moisan, H., Brouillette, E., Jacob, C. L., Langlois-Bégin, P., Michaud, S., and Malouin, F. (2006). Transcription of virulence factors in *Staphylococcus aureus* small-colony variants isolated from cystic fibrosis patients is influenced by SigB. *J. Bacteriol.* 188(1), 64-76.

Patel, D., Kosmidis, C., Seo, S. M., and Kaatz, G. W. (2010). Ethidium bromide MIC screening for enhanced efflux pump gene expression or efflux activity in *Staphylococcus aureus*. *Antimicrob. Agents Chemother.* 54(12), 5070-5073.

Zhao, L., Xue, T., Shang, F., Sun, H., and Sun, B. (2010). *Staphylococcus aureus* AI-2 quorum sensing associates with the KdpDE two-component system to regulate capsular polysaccharide synthesis and virulence. *Infect. Immun.* 78(8), 3506-3515.
